# Supplementary material for: Ube3a unsilencer for the potential treatment of Angelman syndrome
Source: Nat Commun. 2024 Jul 8;15:5558. doi: 10.1038/s41467-024-49788-8 (PMC11231141; doi:10.1038/s41467-024-49788-8)
Supplement: Supplementary file 1 — Supplementary Information [file 41467_2024_49788_MOESM1_ESM.pdf]

Supplementary Information

(Supplementary Figures, Supplementary Table, and Supplementary References)

Ube3a unsilencer for the potential treatment of Angelman syndrome

Hanna Vihma[1], Kelin Li[2,*], Anna Welton-Arndt[3,*], Audrey L. Smith[1], Kiran R. Bettadapur[1], Rachel B. Gilmore[4], Eric Gao[1], Justin L. Cotney[4], Hsueh-Cheng Huang[5], Jon L. Collins[6], Stormy J. Chamberlain[4], Hyeong-Min Lee[1,†,#], Jeffrey Aubé[2,3,#], and Benjamin D. Philpot[1,#]

[1]Department of Cell Biology & Physiology, Neuroscience Center, and Carolina Institute for Developmental Disabilities, University of North Carolina at Chapel Hill, NC, USA

[2]Division of Chemical Biology and Medicinal Chemistry, UNC Eshelman School of Pharmacy, University of North Carolina at Chapel Hill, NC, USA

[3]Department of Chemistry, University of North Carolina at Chapel Hill, NC, USA

[4]Department of Genetics and Genome Sciences, University of Connecticut School of Medicine, Farmington, CT USA

[5]Deerfield Discovery and Development, Deerfield Management, New York, NY, USA

[6]Office of the Vice Chancellor for Research, University of North Carolina at Chapel Hill at Chapel Hill, NC, USA

[*]These authors contributed equally

[†]Current address: Department of Biochemistry and Molecular Biology, Hollings Cancer Center, Medical University of South Carolina. Charleston, SC USA

[#]Co-corresponding authors: bphilpot@med.unc.edu, jaube@email.unc.edu, dr.hmlee@gmail.com

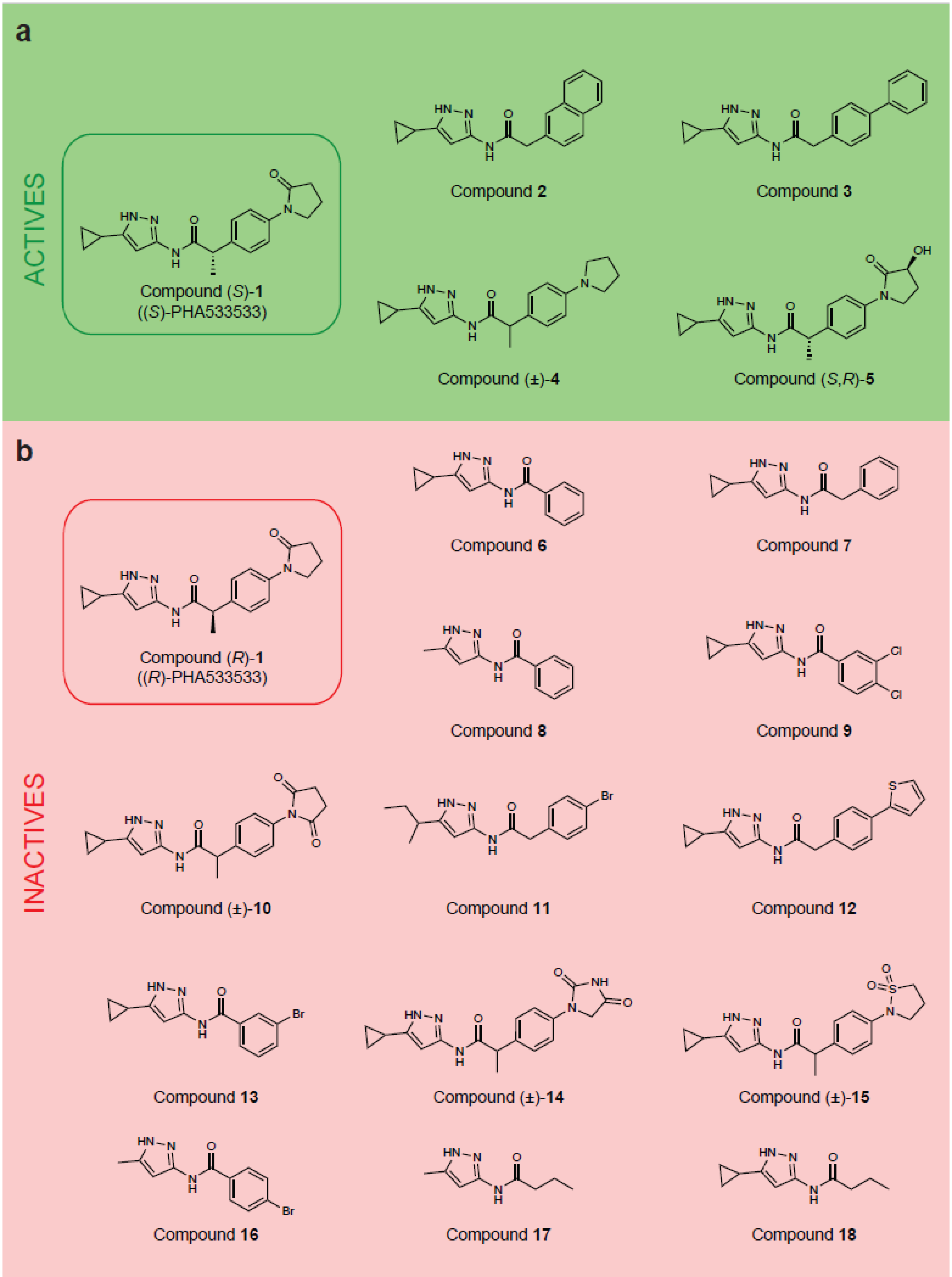

Supplementary Figure 1. Molecular structures of (*S*)- and (*R*)-PHA533533 and seventeen PHA533533 analogs tested for their capability to unsilence paternal *Ube3a*. **(a)** (*S*)-PHA533533 ((*S*)-**1**) and four other active analogs of (*S*)-PHA533533. **(b)** (*R*)-PHA533533 ((*R*)-**2**) and thirteen analogs that lack activity for unsilencing paternal *Ube3a*. Compounds **2**, **6**, **7**, **8**, **9**, **11**, **12**, **13**, **16**, **17**, **18** have been previously published in Pevarello et al., 2004[1], compounds (*S*)-**1**, (*R*)-**1**, **3**, (±)-**4**, (±)-**10**, (±)-**14**, (±)-**15** have been published in Pevarello et al., 2005[2], and compound (*S*,*R*)-**5** in Nesi et all., 2006[3].

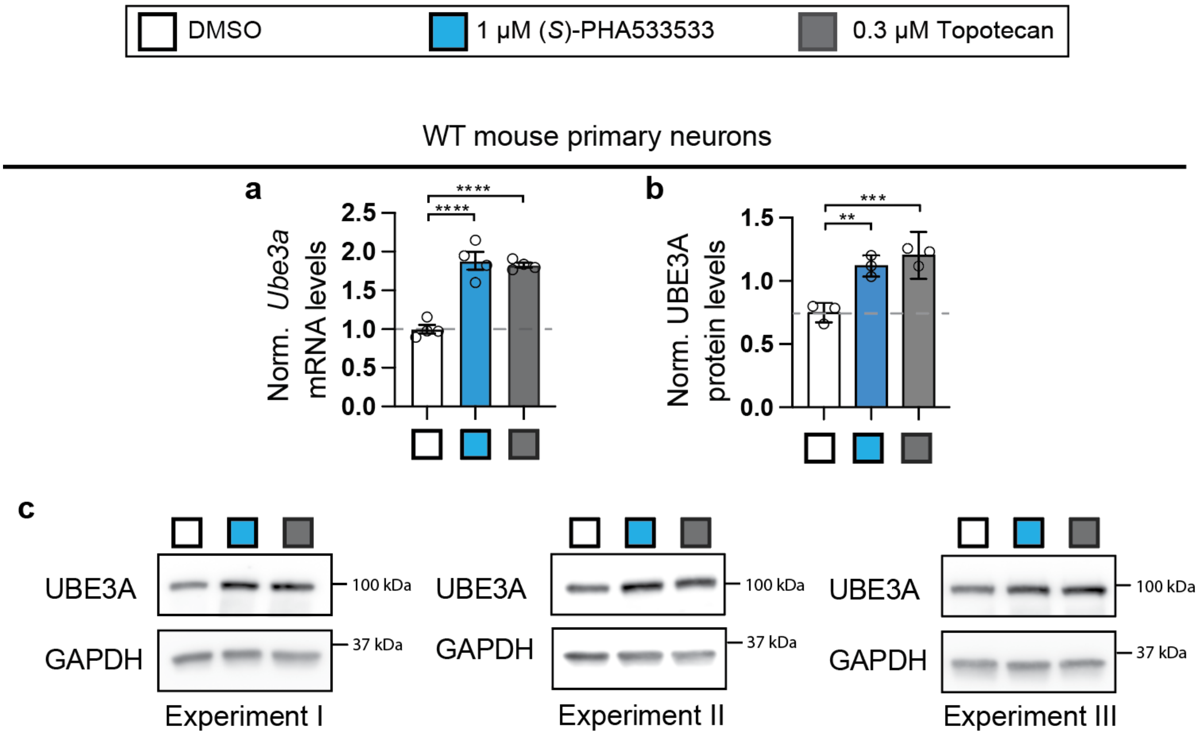

Supplementary Figure 2. (*S*)-PHA533533 and topotecan produce *Ube3a* mRNA and UBE3A protein from the paternal allele in mouse wild-type primary neurons. Primary cortical neurons derived from wild-type (WT) mice were treated either with 0.1% DMSO as a vehicle, 1 µM (*S*)-PHA533533, or 0.3 µM topotecan for 72 hours at DIV7. Following the treatment, the relative quantity of **(a)** *Ube3a* transcript was determined by quantitative RT-PCR, and the expression of **(b)** UBE3A protein was determined through western blotting, with the data averaged across three individual experiments, depicted in **(c)**. All data were normalized to GAPDH/*Gapdh* expression, log-transformed, mean-centered, and autoscaled for statistical analysis (n = 4 experiments for RT-PCR, and n = 3 for western blot; mean ± SEM, one-way ANOVA followed by Dunnett's *post hoc* test, ** $p < 0.01$, *** $p < 0.001$, **** $p < 0.0001$). µM – micromolar; DMSO – dimethyl sulfoxide; kDA – kilodalton; Norm. – normalized. Source data and comprehensive statistics, including F-values, degrees of freedom, confidence intervals, and exact p-values, are provided as a Source Data file.

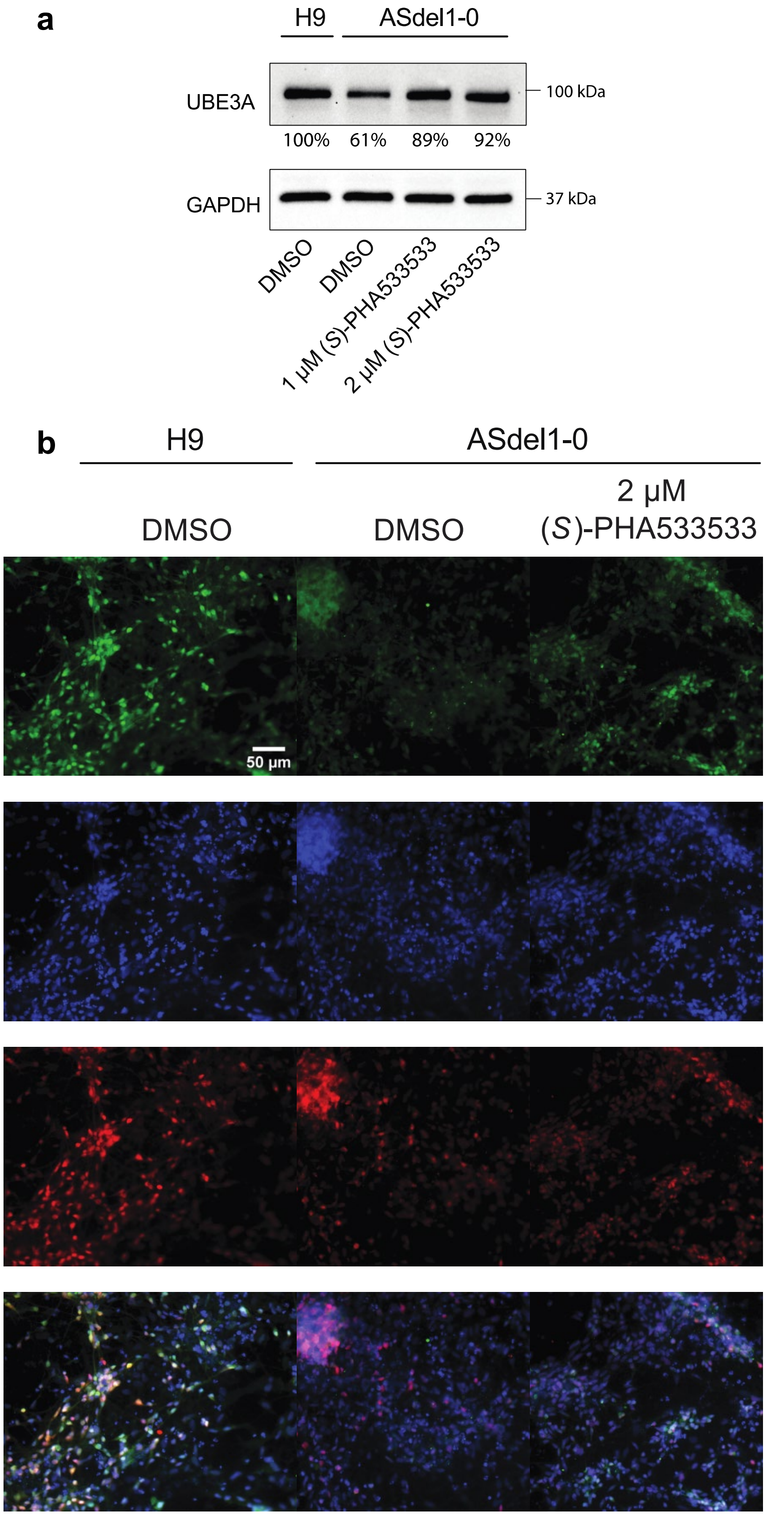

Supplementary Figure 3. (*S*)-PHA533533 treatment increases UBE3A protein levels in human neurons derived from an Angelman syndrome patient. Human neurons differentiated from either H9 hESC control cell line or ASdel1-0 iPSCs for 10 weeks under the same conditions were treated with 0.1% DMSO (vehicle), or 1 or 2 µM (*S*)-PHA533533 for 72 hours, as indicated. Example showing **(a)** the relative quantity of UBE3A protein was determined through western blotting, and **(b)** immunofluorescent images of fixed cells stained for UBE3A, NeuN, and DAPI. µM – micromolar; DAPI – 4′,6-diamidino-2-phenylindole, DMSO – dimethyl sulfoxide; kDA – kilodalton; Norm. – normalized. Source data are provided as a Source Data file.

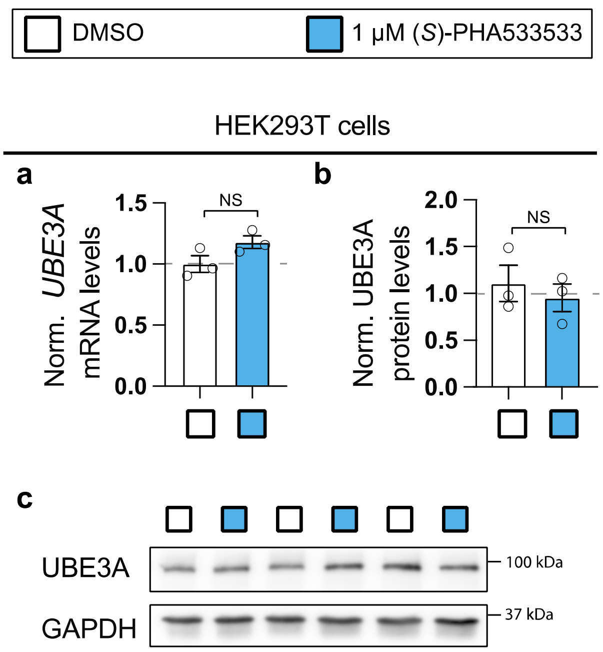

Supplementary Figure 4. (*S*)-PHA533533 treatment does not change *UBE3A* mRNA and protein levels in non-neuronal cells that express *UBE3A* biallelically. Human HEK293T cells were treated either with 0.1% DMSO (vehicle) or 1 µM (*S*)-PHA533533 for 48 hours. Following the treatment, the relative quantity of **(a)** *UBE3A* transcript was determined by quantitative RT-PCR, and the expression of **(b)** UBE3A protein was determined through western blotting, with the data averaged from three individual wells per treatment, as depicted in **(c)**. All data were normalized to GAPDH/*GAPDH* expression, log-transformed, mean-centered, and autoscaled for statistical analysis (n = 3; mean ± SEM, two-tailed T-test, NS = non-significant). µM – micromolar; Norm. – normalized; DMSO – dimethyl sulfoxide; kDA – kilodalton. Source data and comprehensive statistics, including F-values, degrees of freedom, confidence intervals, and exact p-values, are provided as a Source Data file.

Supplementary Table 1. Small molecule screening data

Category	Parameter	Description
Assay	Type of assay	In vitro cell based assay
	Target	Reporter UBE3A-YFP
	Primary measurement	Detection of fluorescence
	Key reagents	Standard primary neuronal culture, anti-GFP antibody (Novus Cat# NB600-308), anti-rabbit Alexa Fluor 488 antibody (Molecular Probes Cat# A-11008)
	Assay protocol	Refer to the Methods (Immunofluorescence, high-content imaging, and dose-response analyses)
	Additional comments	
Library	Library size	~2800 small molecules
	Library composition	Annotated bioactive compounds
	Source	Pfizer
	Additional comments	Jones, L. H. & Bunnage, M. E. Applications of chemogenomic library screening in drug discovery. *Nat. Rev. Drug Discov.* **16**, 285–296 (2017)[4].
Screen	Format	384-well plate (4 wells/compounds)
	Concentration(s) tested	1 μM, 0.1% DMSO
	Plate controls	DMSO or topotecan treated well in every assay plate
	Reagent/ compound dispensing system	Manual (no automation)
	Detection instrument and software	BD Pathway 855, CellProfiler 2.1.1
	Assay validation/QC	Yes/no type screen with hit criteria (relative intensity to compared to the topotecan-treated)
	Correction factors	N/A
	Normalization	Normalized to negative & positive controls (DMSO or topotecan-treated)
	Additional comments	
Post-HTS analysis	Hit criteria	Relative intensity [> 1.25] to compared to the topotecan-treated
	Hit rate	~0.071%
	Additional assay(s)	Re-screen, dose-response test, analog test, orthogonal test
	Confirmation of hit purity and structure	Resynthesis of the chiral forms, (*R*) and (*S*) (~98%)
	Additional comments	

Supplementary References

1. Pevarello, P. *et al.* 3-Aminopyrazole inhibitors of CDK2/cyclin A as antitumor agents. 1. Lead finding. *J. Med. Chem.* **47**, 3367–3380 (2004).
2. Pevarello, P. *et al.* 3-Aminopyrazole Inhibitors of CDK2/Cyclin A as Antitumor Agents. 2. Lead Optimization. *J. Med. Chem.* **48**, 2944–2956 (2005).
3. Nesi, M., Borghi, D., Brasca, M. G., Fiorentini, F. & Pevarello, P. A practical synthesis of the major 3-hydroxy-2-pyrrolidinone metabolite of a potent CDK2/cyclin A inhibitor. *Bioorg. Med. Chem. Lett.* **16**, 3205–3208 (2006).
4. Jones, L. H. & Bunnage, M. E. Applications of chemogenomic library screening in drug discovery. *Nat. Rev. Drug Discov.* **16**, 285–296 (2017).
