# Supplementary material for: Ube3a unsilencer for the potential treatment of Angelman syndrome
Source: Nat Commun. 2024 Jul 8;15:5558. doi: 10.1038/s41467-024-49788-8 (PMC11231141; doi:10.1038/s41467-024-49788-8)
Supplement: Supplementary file 3 — Reporting Summary [file 41467_2024_49788_MOESM3_ESM.pdf]

nature portfolio

Corresponding author(s): Benjamin D. Philpot, Jeffrey Aubé, Hyeong-Min Lee

Last updated by author(s): Mar 27, 2024

Reporting Summary

Nature Portfolio wishes to improve the reproducibility of the work that we publish. This form provides structure for consistency and transparency in reporting. For further information on Nature Portfolio policies, see our Editorial Policies and the Editorial Policy Checklist.

Statistics

For all statistical analyses, confirm that the following items are present in the figure legend, table legend, main text, or Methods section.

n/a	Confirmed	
☐	☒	The exact sample size (n) for each experimental group/condition, given as a discrete number and unit of measurement
☐	☒	A statement on whether measurements were taken from distinct samples or whether the same sample was measured repeatedly
☐	☒	The statistical test(s) used AND whether they are one- or two-sided<br>*Only common tests should be described solely by name; describe more complex techniques in the Methods section.*
☒	☐	A description of all covariates tested
☐	☒	A description of any assumptions or corrections, such as tests of normality and adjustment for multiple comparisons
☐	☒	A full description of the statistical parameters including central tendency (e.g. means) or other basic estimates (e.g. regression coefficient) AND variation (e.g. standard deviation) or associated estimates of uncertainty (e.g. confidence intervals)
☐	☒	For null hypothesis testing, the test statistic (e.g. F, t, r) with confidence intervals, effect sizes, degrees of freedom and P value noted<br>*Give P values as exact values whenever suitable.*
☒	☐	For Bayesian analysis, information on the choice of priors and Markov chain Monte Carlo settings
☒	☐	For hierarchical and complex designs, identification of the appropriate level for tests and full reporting of outcomes
☒	☐	Estimates of effect sizes (e.g. Cohen's d, Pearson's r), indicating how they were calculated

Our web collection on statistics for biologists contains articles on many of the points above.

Software and code

Policy information about availability of computer code

Data collection	All data were collected with commercially available equipment, as described in manuscript.
Data analysis	All data were analyzed with commercially available software as described in manuscript.

For manuscripts utilizing custom algorithms or software that are central to the research but not yet described in published literature, software must be made available to editors and reviewers. We strongly encourage code deposition in a community repository (e.g. GitHub). See the Nature Portfolio guidelines for submitting code & software for further information.

Data

Policy information about availability of data

All manuscripts must include a data availability statement. This statement should provide the following information, where applicable:

- Accession codes, unique identifiers, or web links for publicly available datasets
- A description of any restrictions on data availability
- For clinical datasets or third party data, please ensure that the statement adheres to our policy

All data underlying this manuscript are available in the Source Data file.

Research involving human participants, their data, or biological material

Policy information about studies with human participants or human data. See also policy information about sex, gender (identity/presentation), and sexual orientation and race, ethnicity and racism.

Reporting on sex and gender	N/A
Reporting on race, ethnicity, or other socially relevant groupings	N/A
Population characteristics	N/A
Recruitment	N/A
Ethics oversight	N/A

Note that full information on the approval of the study protocol must also be provided in the manuscript.

Field-specific reporting

Please select the one below that is the best fit for your research. If you are not sure, read the appropriate sections before making your selection.

☒ Life sciences ☐ Behavioural & social sciences ☐ Ecological, evolutionary & environmental sciences

For a reference copy of the document with all sections, see nature.com/documents/nr-reporting-summary-flat.pdf

Life sciences study design

All studies must disclose on these points even when the disclosure is negative.

Sample size	No statistical methods were used to predetermine sample size for experiments. The sample sizes were based on those generally employed in the field. All the data include at least three biological replicates.
Data exclusions	All the data were included in the analyses.
Replication	All experiments, with the exception of the illustrative data depicted in Supplementary Figure 3, were conducted in a minimum of three biological replicates. All findings were successfully replicated or reproduced at least three times in independent experiments.
Randomization	Randomization is not applicable for the experiments in this study.
Blinding	The investigators were not blinded since analyses relied on unbiased measurements of quantitative parameters. Standardized procedures for data collection and analysis were used to prevent bias.

Reporting for specific materials, systems and methods

We require information from authors about some types of materials, experimental systems and methods used in many studies. Here, indicate whether each material, system or method listed is relevant to your study. If you are not sure if a list item applies to your research, read the appropriate section before selecting a response.

Materials & experimental systems

n/a	Involved in the study	
☐	☒	Antibodies
☐	☒	Eukaryotic cell lines
☒	☐	Palaeontology and archaeology
☐	☒	Animals and other organisms
☒	☐	Clinical data
☒	☐	Dual use research of concern
☒	☐	Plants

Methods

n/a	Involved in the study	
☒	☐	ChIP-seq
☒	☐	Flow cytometry
☒	☐	MRI-based neuroimaging

Antibodies

Antibodies used	rabbit anti-GFP antibody (NB 600308, Novus Biologicals), anti-rabbit Alexa Fluor 488 antibody (A11008, Thermo Fisher), anti-mouse Alexa Fluor 488 antibody (A11029, Thermo Fisher), goat anti-rabbit Alexa Fluor 594 (A11012, ThermoFisher), DAPI (D1306 and

	P36941, Invitrogen), mouse anti-NeuN antibody (MAB 377, Millipore, and ab177487, Abcam), anti-mouse Alexa Fluor 568 antibody (A21124, Thermo Fisher), anti-GFP (NB600-303, Novus Biologicals), anti-CDK5 (sc-6247, Santa Cruz Biotechnology), anti-CDK2 (ab64669, Abcam), anti-TOP1 (sc-271285, Santa Cruz Biotechnology), anti-GAPDH (MAB374, Millipore Sigma), anti-b-Tubulin (ab6046, Abcam), and anti-b-Actin (A1978, Millipore Sigma). HRP-conjugated secondary antibodies (31430 and 31460, Invitrogen, and 7076, Cell Signaling), mouse anti-UBE3A (SAB1404508 and E8655, Sigma Millipore), guinea pig anti-NeuN (ABN90, Sigma Millipore), goat anti-mouse Alexa Fluor 488 (A21131, Invitrogen) and anti-guinea pig Cy3 (706-165-148, Jackson ImmunoResearch Inc.)
Validation	All antibodies were characterized/validated by the commercial vendor or previously published manuscripts.

Eukaryotic cell lines

Policy information about cell lines and Sex and Gender in Research

Cell line source(s)	The ASdel1-0 lines were reprogrammed at UConn Health. The publication describing that process is Chamberlain et al 2010 doi: 10.1073/pnas.1004487107. The I-P edit in the ASdel1-0 line was generated at UConn Health. The paper describing that process is Hsiao et al 2019 doi: 10.1073/pnas.1815279116. iPSC cell lines are available upon reasonable request and after completion of Material Transfer Agreements through the University of Connecticut Cell and Genome Engineering Core.<br><br>H9 wild type ESCs were purchased from WiCell Research Institute.<br><br>HEK293T cells were purchased from ATCC.
Authentication	None of the cell lines were authenticated as part of this study, but were described in previous publications.
Mycoplasma contamination	The cell lines were not tested for mycoplasma during this study.
Commonly misidentified lines (See ICLAC register)	The cell line used in this study is not present in the registry of commonly misidentified lines.

Animals and other research organisms

Policy information about studies involving animals; ARRIVE guidelines recommended for reporting animal research, and Sex and Gender in Research

Laboratory animals	Wild-type, Ube3a(m-/p+), Ube3a(m+/pYFP) mice on C57BL/6 background were used in this paper. Mice up 5-month-old were used.
Wild animals	N/A
Reporting on sex	Sex of the mice was not considered as a biological variable.
Field-collected samples	N/A
Ethics oversight	All animal related experiments follow the UNC IACUC guildlines, and approved animal handling protocols #20-156 and #23-138 granted to Ben Philpot lab at UNC

Note that full information on the approval of the study protocol must also be provided in the manuscript.

Plants

Seed stocks	N/A
Novel plant genotypes	N/A
Authentication	N/A
